# New records of birds from Central Vietnam

**DOI:** 10.3897/BDJ.12.e133721

**Published:** 2024-08-20

**Authors:** Hung Manh Le, Thang Ho, Trung Nguyen Thoi Le, Hoa Ngoc Nguyen, Thach Dang Nguyen

**Affiliations:** 1 Institute of Ecology and Biological Resources, Vietnam Academy of Science and Technology, 18 Hoang Quoc Viet Street, Cau Giay District,, Hanoi, Vietnam Institute of Ecology and Biological Resources, Vietnam Academy of Science and Technology, 18 Hoang Quoc Viet Street, Cau Giay District, Hanoi Vietnam; 2 Thua Thien Hue Department of Science and Technology, Vo Nguyen Giap Street, Xuan Phu commune, Hue, Vietnam Thua Thien Hue Department of Science and Technology, Vo Nguyen Giap Street, Xuan Phu commune Hue Vietnam; 3 Central Coast Nature Museum, No.7 Vy Da Street, Vy Da commune, Hue, Vietnam Central Coast Nature Museum, No.7 Vy Da Street, Vy Da commune Hue Vietnam

**Keywords:** Ornithology, biodiversity, Annam, birds, taxonomy, morphology, range extensions.

## Abstract

**Background:**

There has been a series of bird surveys conducted in Vietnam over the last 20 years. However, most of these studies and surveys have focused on sites in Tonkin, the Red River Delta, Cochinchina (Mekong Delta), the Central Highlands and mountainous areas of Central Annam (Central region of Vietnam). The central coastal plain as well as the mountainous region of North Annam have rarely been comprehensively investigated.

**New information:**

As a result of our field surveys in 2023 and 2024, a total of 15 species of birds are recorded for the first time from North, Central and South Annam, comprising one Frigate-Bird species (Fregatidae), one ibis (Threskiornithidae), one reed-warbler (Acrocephalidae), one Treecreeper (Certhiidae), two buntings (Emberizidae), one chat (Muscicapidae), one yuhina (Zosteropidae), two nuthatches (Sittidae), two members of the laughingthrush family (Leiothrichidae) and three bulbuls (Pycnonotidae). In addition to photographs confirming the new records, we provide information on the distribution and conservation status of these newly-recorded bird species from Central Vietnam.

## Introduction

Vietnam is identified as one of the avifaunistically most diverse countries in Southeast Asia (Craik and Minh 2018). Vietnam falls within Indo-Burma, a globally important hotspot for biodiversity and hosts a large number of rare and endemic species (Sterling et al. 2006). To date, a total of 63 Important Bird Areas (IBA) (Tordoff et al. 2002) and 102 Key Biodiversity Areas (KBA) have been identified in Vietnam (Tordoff et al. 2012). Over the past 30 years, the number of Vietnamese bird species has risen significantly due to an increase in avifaunal surveys as well as the number of researchers, birdwatchers and wildlife photographers. The number of bird species recorded in Vietnam in 1995 amounted to 828 and rose to 918 species in 2021 (Quy and Cu 1995, Cu et al. 2000, Robson 2008, Hung 2012, Craik and Minh 2018, Hung et al. 2021b). Many species have undergone range extensions as our knowledge of their distribution has become better over the past ten years (Hung et al. 2014, Hung et al. 2016, Hung and Quyet 2017, Hung et al. 2017a, Hung et al. 2017b, Hung et al. 2017c, Hung et al. 2019, Hung et al. 2020, Hung et al. 2021).

As a result of our recent field surveys in the wetland areas along the central coastal plain (Thua Thien Hue, Da Nang, Quang Nam, Quang Ngai, Binh Dinh, Khanh Hoa Provinces) and Na Ngoi Commune, Ky Son District Nghe An Province, we herein report 15 new records from central Vietnam.

## Materials and methods

**Study areas**

**Figure 1.**
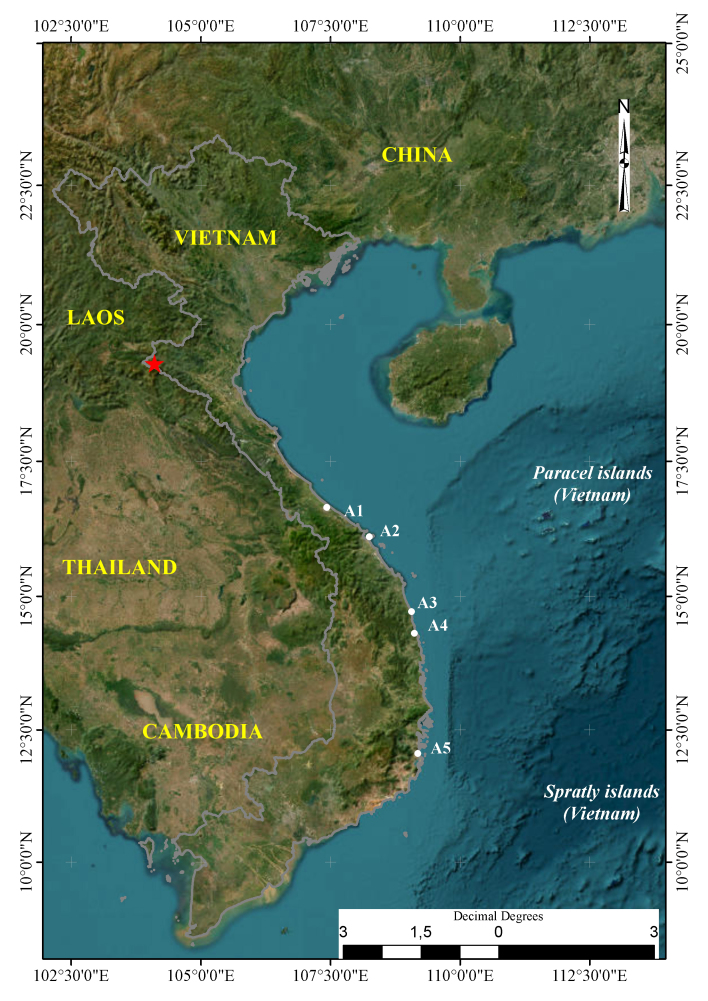
Map showing the survey areas: Red star is Ky Son area, Nghe An Province, North Annam: A1 = Tam Giang – Cau Hai Lagoon (Thua Thien Hue Province), A2 = Son Tra Forest and My Khe Beach (Da Nang Province), A3 = An Khe Lake (Quang Ngai Province), A4 = Tra O Lake (Binh Dinh Province), A5 = Thuy Trieu Lake (Khanh Hoa Province).

Field surveys were conducted by Hung Manh Le, Thang Ho, Trung Thoi Nguyen Le, Hoa Ngoc Nguyen and Thach Dang Nguyen (hereafter Hung et al.) from 7 to 21 December 2023 and from 12 to 28 April 2024 in the Provinces of Thua Thien Hue, Da Nang, Quang Nam, Quang Ngai, Binh Dinh and Khanh Hoa in the central coastal plain (from 16°39'052"N 107°25'571"E to 12°03'038"N 109°11'137"E). We also conducted surveys from 22 to 28 December 2023 and from 29 April to 5 May 2024 in the Na Ngoi Commune, Ky Son District, Nghe An Province (from 19°16'307"N 104°11'153"E to 19°13'206"N 104°06'305"E) (now called as Ky Son area) (Fig. 1). Coordinates (WGS 84) and elevations were determined using a GPS Garmin 60CX.

**Figure 2.**
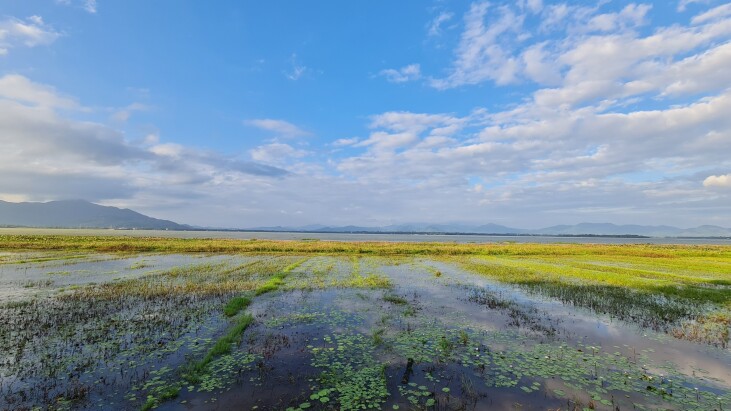
Wetland habitats at Tra O Lake, Binh Dinh Province, Central Annam, Vietnam.

The typical habitat types along the central coastal plain include lagoons (Tam Giang – Cau Hai in Thua Thien Hue Province), coastal beaches (Da Nang and Quang Nam Provinces), freshwater lakes and swamps (Quang Ngai, Binh Dinh and Khanh Hoa Provinces). Surrounding the lagoons and lakes are paddyfields, reeds, grasses, gardens and plantations (Fig. 2).

**Figure 3.**
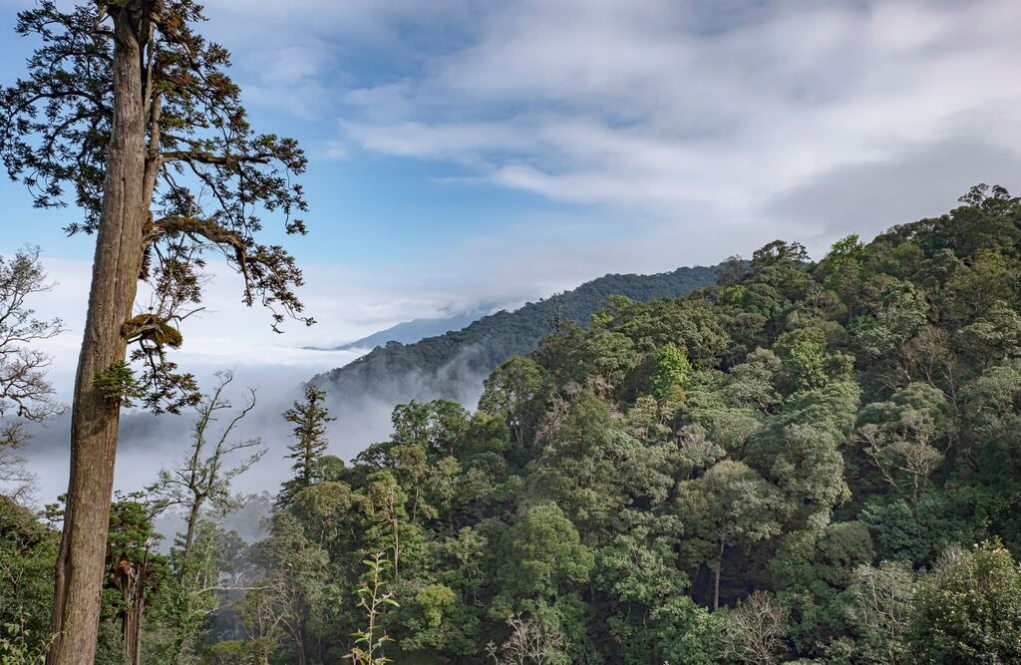
Habitats at Ky Son area, Nghe An Province, North Annam, Vietnam.

The Ky Son area is close to the Laos - Vietnam border (Fig. 1). The typical habitat types in the area include scrub, secondary growth and broadleaved evergreen forest. This area covers an elevation range from 300 to 2,280 m a.s.l. While most of the forest areas from 300 to 1,600 m are highly degraded, the forest from 1,600 to 2,200 m was still fairly intact (Fig. 3).

**Field surveys**

In the central coastal plain areas, we conducted line transects and point counts: Every day (from 5:45 am to 10:00 am and from 2:30 pm to 6:00 pm), the observers walked slowly along the existing trails (around the lagoon, lakes and fishponds) with frequent stops to observe mixed feeding flocks or birds flying over. The observers predominantly focused on key habitats within the survey areas, using the point counts method (Bibby et al. 1998) to count and record all bird species. Boats were also used at some large lakes and lagoons.

For the Ky Son area, only line transect surveys were used. All the bird species and individuals were recorded by direct observation, acoustic recordings and photography. The following equipment was used during the surveys: Swarowski EL 8 x 32 and 10 x 42 binoculars, Swaroski 20 x 80 telescope and field guides for identification (Robson 2008, Hung et al. 2021b), as well as cameras including Canon 1D max III and Nikkon D6 bodies with 500 mm and 600 mm lenses. The conservation status of recorded species follows the IUCN Red List 2024 (IUCN 2024). The ornithological regions in Vietnam follow Robson (2008) and Hung et al. (2021b), which includes six different regions: West Tonkin, East Tonkin, North Annam, Central Annam, South Annam and Cochinchina. Taxonomy follow IOC World Bird List - Version 14.1 (Gill et al. 2024). The diagnoses of 15 species in this study follow Robson (2008).

These records all represent firsts for the region, with none appearing in the most recent reference works (Robson 2008, Hung 2012, Craik and Minh 2018, Hung et al. 2021b). Here, we document fifteen range extensions or regional firsts.

## Checklists

### Taxon treatments

#### Fregata ariel

(Gray, GR, 1845)

FF9DCBD3-A35E-5AD1-9E13-779B41BC753F

https://www.gbif.org/species/2480186

Fregata ariel (Gray, GR, 1845)

##### Materials

- **Type status:** Other material. **Occurrence:** occurrenceID: 6DBE7564-D31F-5DED-91EB-77D0332C6C2B; **Taxon:** kingdom: Animalia; phylum: Chordata; class: Aves; order: Suliformes; family: Fregatidae; genus: Fregata; **Location:** country: Vietnam; stateProvince: Danang; locality: My Khe; verbatimElevation: 30 m; verbatimLatitude: 16°06.353’N; verbatimLongitude: 108°15.513’E

##### Conservation status

Least Concern

##### Distribution

Lesser Frigate-Bird is a scarce non-breeding offshore visitor species in Vietnam, with the only previous records from Cochinchina (Robson 2008, Craik and Minh 2018, Hung et al. 2021b). This is the first confirmed record for the species in Central Annam. Elsewhere, this species has been recorded resident in Australia, Brazil, British Indian Ocean Territory, Brunei Darussalam, China, Christmas Island, Comoros, Fiji, French Polynesia, India, Indonesia, Japan, Kiribati, North Korea, Madagascar, Malaysia, Mauritius, Mayotte, New Caledonia, Philippines, Singapore, Solomon Islands, Sri Lanka, Taiwan, Tanzania, Thailand, Tonga, United States, Vanuatu, breeding in Cocos (Keeling) Islands, Maldives, Marshall Islands, Réunion, Seychelles and non-breeding in Micronesia, northern Mariana Islands, Palau, Timor-Leste, United States Minor Outlying Islands, Djibouti, Eritrea, Israel, Jordan, Kenya, Mozambique, New Zealand, Oman and Somalia (IUCN 2024).

##### Diagnosis

**Figure 4.**
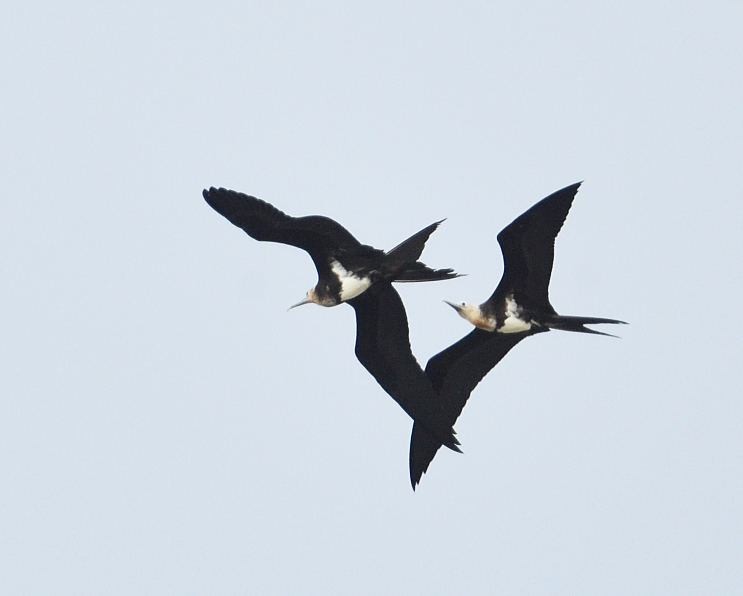
The juvenile individuals of *Fregataariel* (IEBR B.501) were photographed in My Khe Beach, Da Nang City.

Size: 71-81 cm. : Overall blackish plumage with prominent whitish patches extending from side of body to inner underwing-coverts diagnostic. Red gular pouch. : Combination of black hood, belly and lower flanks, white remainder of underparts, extending in axillary spur on to inner underwing-coverts.: shows rufous to brownish-white head, black breast-band, triangular white belly-patch. White axillary spur always present, originating from line of breast-band and angled outwards (towards wing- tip). Band across upperwing-coverts buffish, moderately prominent (Fig. 4)

#### Emberiza pusilla

Pallas, 1776

8E959741-4103-56CF-890D-72B8CA5876C2

https://www.gbif.org/species/2491544

Emberiza pusilla Pallas, 1776

##### Materials

- **Type status:** Other material. **Occurrence:** occurrenceID: E892B0E6-DD9F-5693-8E30-79A648BDF54F; **Taxon:** kingdom: Animalia; phylum: Chordata; class: Aves; order: Passeriformes; family: Emberizidae; genus: Emberiza; **Location:** country: Vietnam; stateProvince: Nghe An; locality: Con Cuong; verbatimElevation: 600 m; verbatimLatitude: 19°17.024’N; verbatimLongitude: 104°10.492’E

##### Conservation status

Least Concern

##### Distribution

Little Bunting has been recorded as a fairly common to common winter visitor in West and East Tonkin (Robson 2008, Craik and Minh 2018, Hung et al. 2021b). This is the first confirmed record for the species in North Annam. Elsewhere, this species has been recorded breeding in Norway, Russian and Sweden, resident in Bangladesh, Bhutan, China, Finland, Hong Kong, India, Japan, Kazakhstan, Kuwait, Laos, Malaysia, Mongolia, Myanmar, North Korea, Nepal, Pakistan, Philippines, Taiwan, Thailand, non-breeding in Iran, Saudi Arabia and vagrant in Afghanistan, Austria, Belgium, Bulgaria, Cyprus, Denmark, Egypt, Estonia, Faroe Islands, France, Germany, Greece, Hungary, Iceland, Ireland, Israel, Italy, Jordan, Latvia, Malta, Netherlands, Poland, Portugal, Slovenia, Spain, Switzerland, Tajikistan, Türkiye, Ukraine, United Kingdom and United States (IUCN 2024).

##### Diagnosis

**Figure 5.**
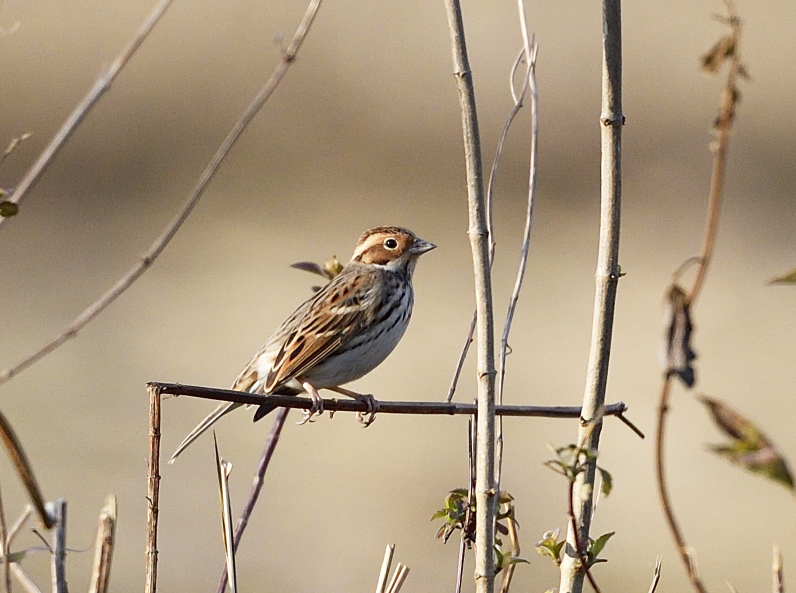
The adult non-breeding of *Emberizapusilla* (IEBR B.536) was photographed in Con Cuong, Nghe An Province.

Size: 12-14 cm. **Adult non-breeding**: Small with finely dark-streaked breast and flanks and rather uniform base colour of upperside. Bold broad blackish (streaky) lateral crown-stripes, eye-stripe and border to rufous-chestnut ear-coverts. Median crown- stripes, supercillium, submoustachial stripe and spot on rear ear-coverts contrastingly buffish, shows rufescent lores and forehead, whitish eye-ring (Fig. 5). **Adult breeding**: Attains diagnostic chestnut flush over most of head, lateral crown-stripes solid black. **Juvenile**: Similar to adult non-breeding. Initially shows much duller lateral crown-stripes, less neat breast and flanks streaking, brower-tinged underparts.

#### Pycnonotus xanthorrhous

Anderson, 1869

1D08363E-9137-5080-A8B9-5C2CE09AEB33

https://www.gbif.org/species/2486130

Pycnonotus xanthorrhous Anderson, 1869

##### Materials

- **Type status:** Other material. **Occurrence:** occurrenceID: 41D184C9-175D-541D-8A7F-13720D77EC32; **Taxon:** kingdom: Animalia; phylum: Chordata; class: Aves; order: Passeriformes; family: Pycnonotidae; genus: Pycnonotus ; **Location:** country: Vietnam; stateProvince: Nghe An; locality: Ky Son; verbatimElevation: 1,658 m; verbatimLatitude: 19°14.207’N; verbatimLongitude: 104°06.542’E

##### Conservation status

Least Concern

##### Distribution

Brown-breasted Bulbul has been recorded as a locally common resident in West Tonkin and East Tonkin (Robson 2008, Craik and Minh 2018, Hung et al. 2021b). This appears to represent the first confirmed record of the species in North Annam, Vietnam. Elsewhere, this species has been recorded breeding in China, Laos, Myanmar and Thailand (IUCN 2024).

##### Diagnosis

**Figure 6.**
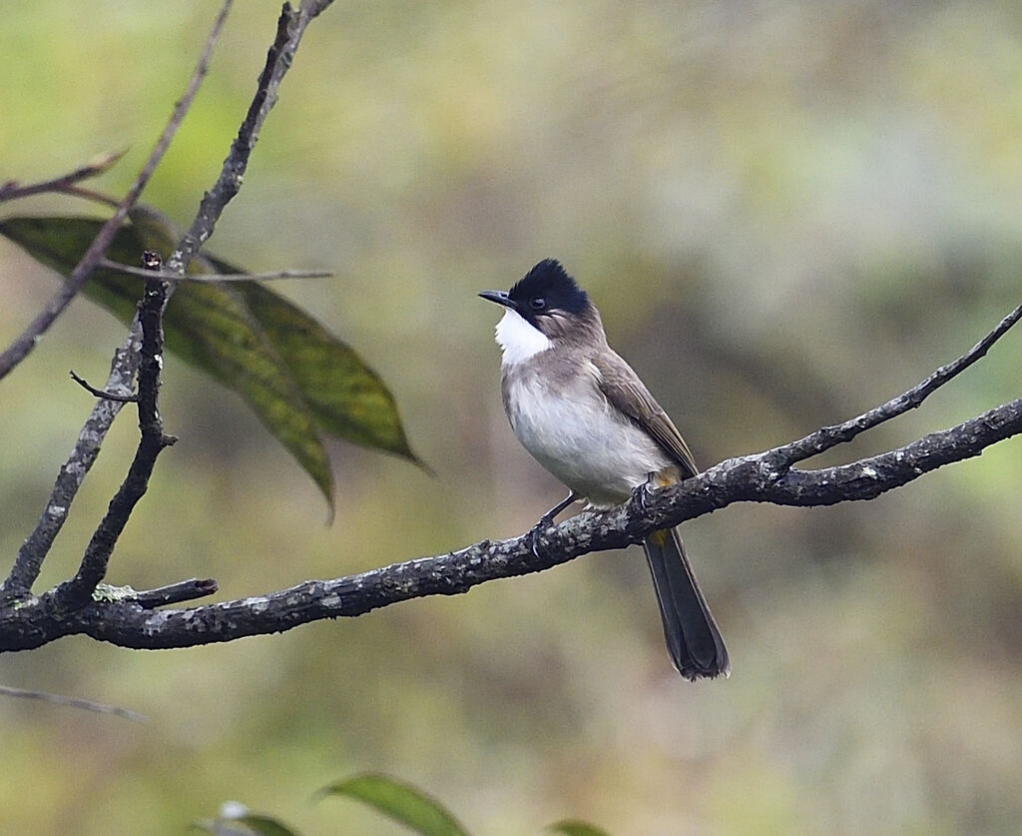
The adult *Pycnonotusxanthorrhous* (IEBR B.554) was photographed in Ky Son area, Nghe An Province.

Size: 20 cm. **Adult**: Brown upperparts, black cap and cheeks, short crest, brown ear-coverts and breast-band, white throat, plain pale greyish underparts, deep yellow undertail-coverts, little or no white on tail-tip (Fig. 6). **Juvenile**: Breast-band less distinct, dark head markings browner, paler underparts.

#### Alcurus striatus

(Blyth, 1842)

ACD91A2B-148F-5B08-9F0E-DD6856BF17D5

https://www.gbif.org/species/10943689

##### Materials

- **Type status:** Other material. **Occurrence:** occurrenceID: 168CB646-1B88-56B9-94FB-1F4797587B4B; **Taxon:** kingdom: Animalia; phylum: Chordata; class: Aves; order: Passeriformes; family: Pycnonotidae; genus: Alcurus ; **Location:** country: Vietnam; stateProvince: Nghe An; locality: Ky Son; verbatimElevation: 1,946 m; verbatimLatitude: 19°13.391’N; verbatimLongitude: 104°06.145’E

##### Conservation status

Least Concern

##### Distribution

Striated Bulbul has been recorded as a uncommon resident in West Tonkin (Robson 2008, Craik and Minh 2018, Hung et al. 2021b). This is the first confirmed record of the species for North Annam. Elsewhere, this species has been recorded breeding in Bhutan, China, India, Laos, Myanmar, Nepal and Thailand (IUCN 2024).

##### Diagnosis

**Figure 7.**
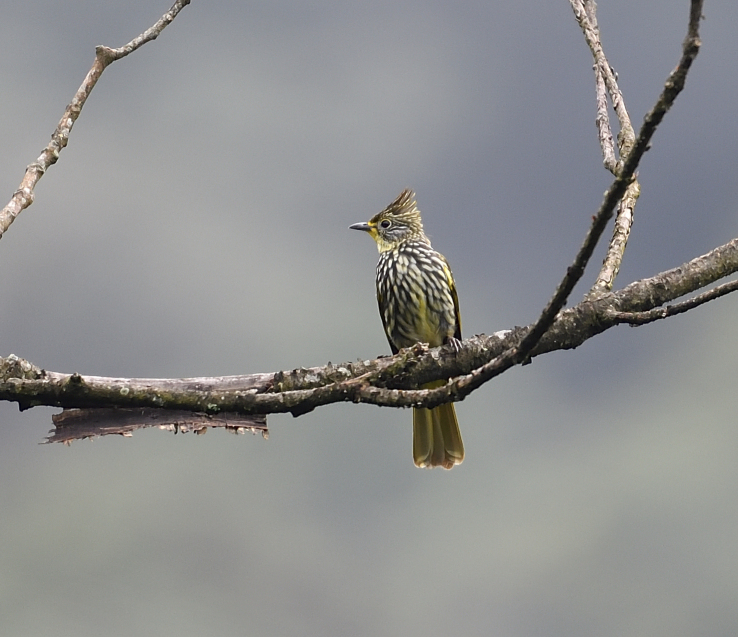
The adult of *Alcurusstriatus* (IEBR B.569) was photographed in Ky Son area, Nghe An Province.

Size: 23 cm. **Adult**: Combination of heavy yellowish-white streaking on head and body, prominent crest and yellow spectacles, mostly yellow throat and undertail-coverts (Fig. 7).

#### Spizixos canifrons

Blyth, 1845

2EBB3AD3-48CB-538F-844B-A4C1BE5CB78A

https://www.gbif.org/species/2486221

Spizixos canifrons Blyth, 1845

##### Materials

- **Type status:** Other material. **Occurrence:** occurrenceID: DC8BCA75-3684-55D4-B7A6-74D2E3414F55; **Taxon:** kingdom: Animalia; phylum: Chordata; class: Aves; order: Passeriformes; family: Pycnonotidae; genus: Spizixos ; **Location:** country: Vietnam; stateProvince: Nghe An; locality: Ky Son; verbatimElevation: 1,923 m; verbatimLatitude: 19°13.476’N; verbatimLongitude: 104°06.109’E

##### Conservation status

Least Concern

##### Distribution

Crested Finchbill has been recorded as a common resident in West Tonkin (Robson 2008, Craik and Minh 2018, Hung et al. 2021b). This is the first confirmed record of the species for North Annam, Central Vietnam. Elsewhere, this species has been recorded as a resident in Bangladesh, China, India, Laos, Myanmar and Thailand (IUCN 2024).

##### Diagnosis

**Figure 8.**
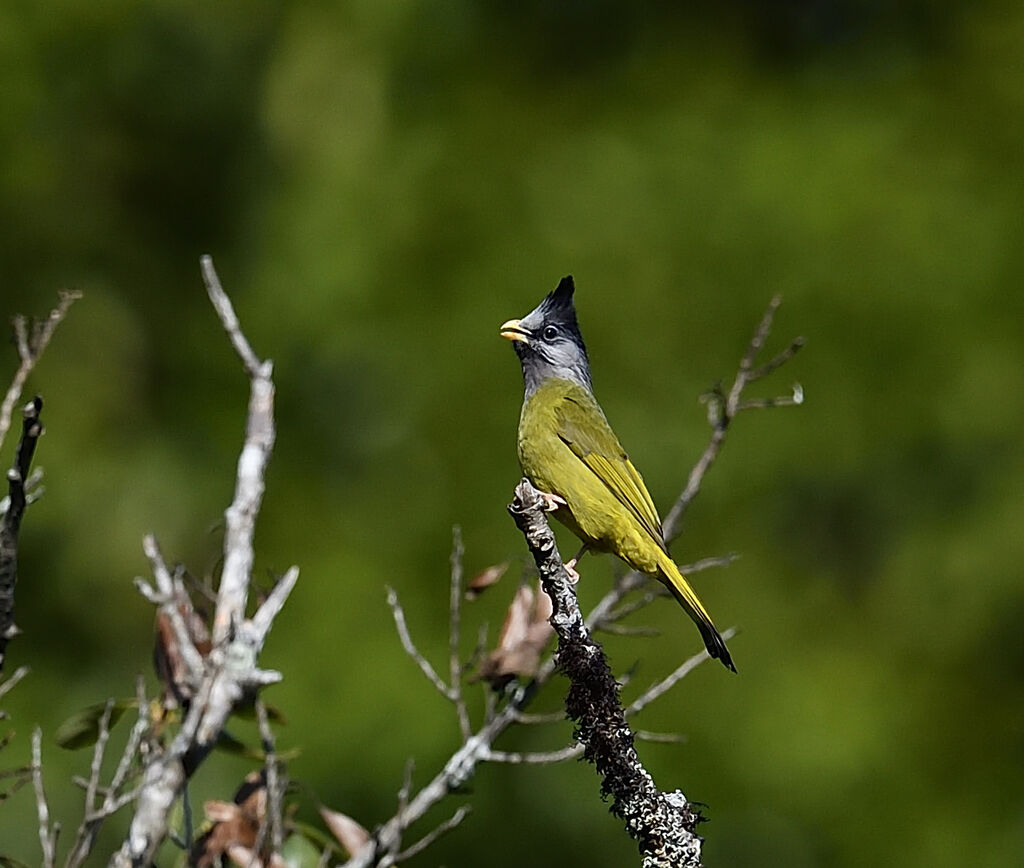
The adult of *Spizixoscanifrons* (IEBR B.579) was photographed in the Ky Son area, Nghe An Province.

Size: 21.5 cm. **Adult**: Greenish to yellowish-green plumage, thick pale yellowish bill and erect pointed crest distinctive. Head mostly greyish with blacker lores, crest and throat, blackish tail-tip (Fig. 8). **Juvenile**: Crown and crest paler and mixed with green, yellowish-green forehead and throat, paler ear-coverts, dark tail-band less distinct.

#### Saxicola jerdoni

(Blyth, 1867)

6BB8888E-F9D8-592F-A4D0-B77F0654902B

https://www.gbif.org/species/2492520

Saxicola jerdoni (Blyth, 1867)

##### Materials

- **Type status:** Other material. **Occurrence:** occurrenceID: 0BDC36E8-433C-5B6D-BCBB-417BE007BD51; **Taxon:** kingdom: Animalia; phylum: Chordata; class: Aves; order: Passeriformes; family: Muscicapidae; genus: Saxicola ; **Location:** country: Vietnam; stateProvince: Nghe An; locality: Ky Son; verbatimElevation: 1,043 m; verbatimLatitude: 19°14.471’N; verbatimLongitude: 104°08.129’E

##### Conservation status

Least Concern

##### Distribution

Jerdon's Bushchat has been recorded as a rare to scarce resident in East Tonkin, with additional records of uncertain status in West Tonkin (Robson 2008, Craik and Minh 2018, Hung et al. 2021b). This is the first confirmed record of the species for North Annam, Central Vietnam. Elsewhere, this species has been recorded as resident in Nepal, Bangladesh, China, India, Laos and Thailand (IUCN 2024).

##### Diagnosis

**Figure 9.**
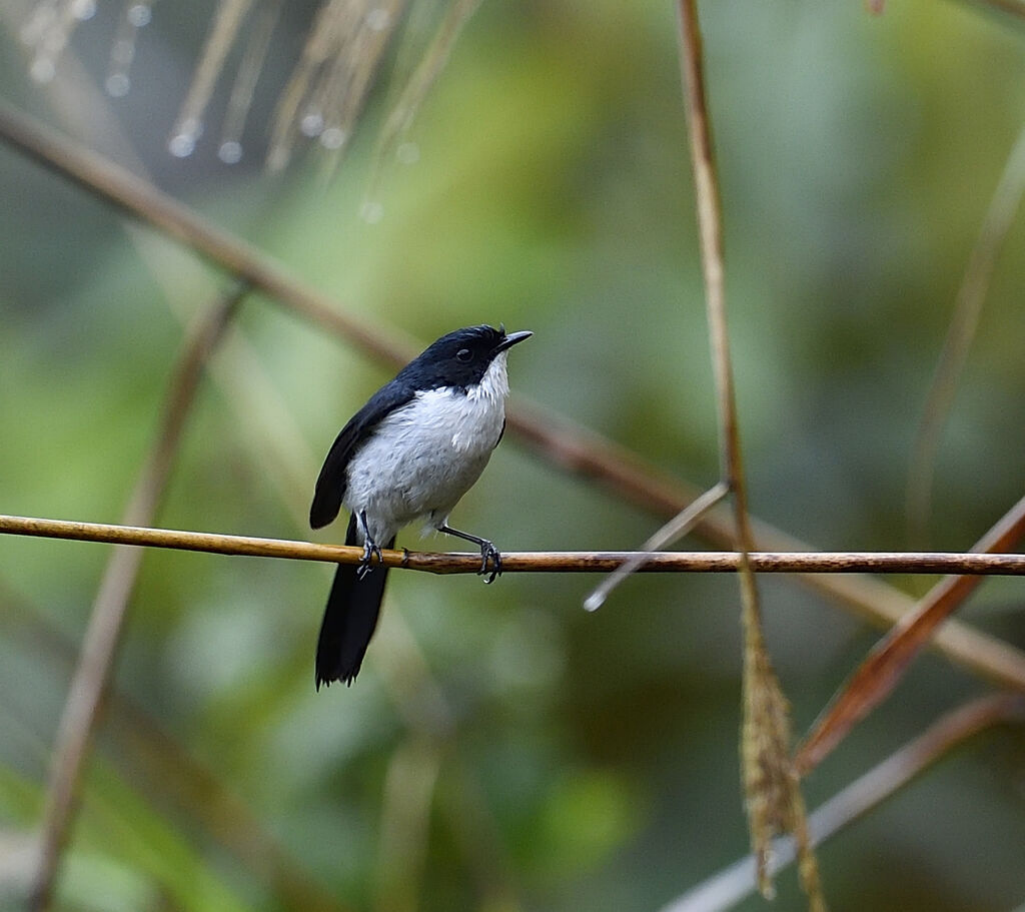
The male *Saxicolajerdoni* (IEBR B.582) was photographed in the Ky Son area, Nghe An Province.

**Figure 10.**
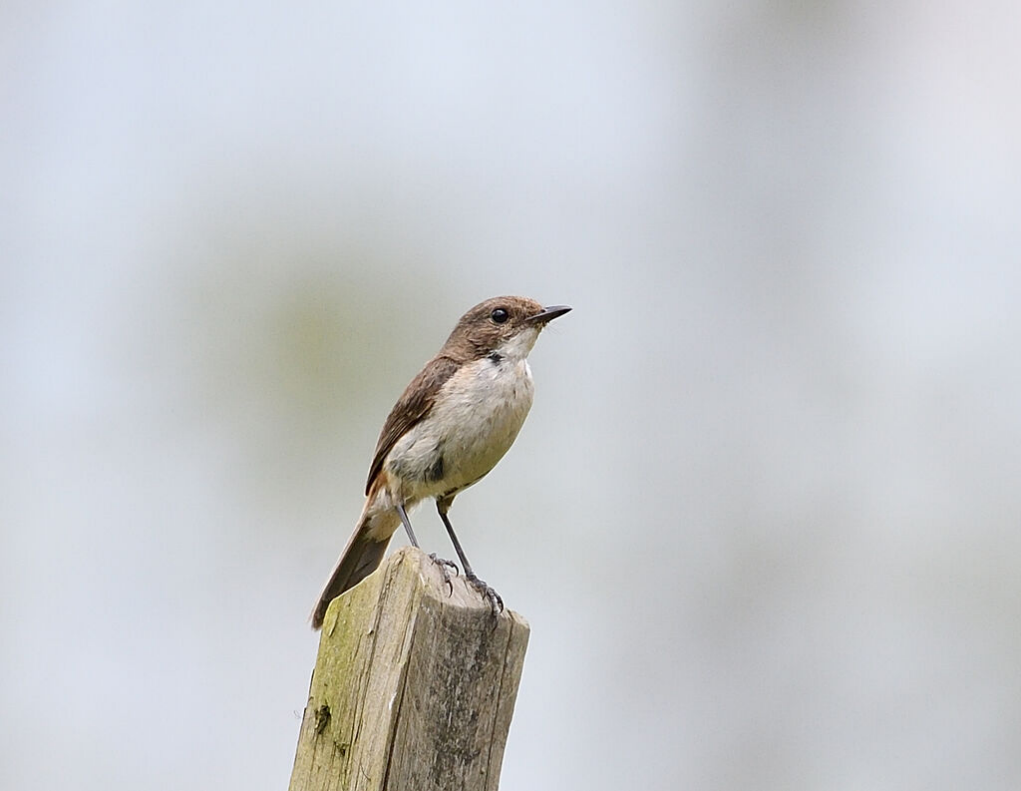
The female of *Saxicolajerdoni* (IEBR B.583) was photographed in the Ky Son area, Nghe An Province.

Size: 15 cm. **Male**: Uniformly glossy blackish upperside and all white underside diagnostic (Fig. 9). **Female**: Brown upperparts, greyish underparts, whiter on throat (Fig. 10).

#### Yuhina gularis

Hodgson, 1836

45BB8F2D-9E2E-5C40-B083-AF3E2C51C574

https://www.gbif.org/species/2493181

Yuhina gularis Hodgson, 1836

##### Materials

- **Type status:** Other material. **Occurrence:** occurrenceID: 393410CA-23A0-5033-A3F1-BB2784475B41; **Taxon:** kingdom: Animalia; phylum: Chordata; class: Aves; order: Passeriformes; family: Zosteropidae; genus: Yuhina ; **Location:** country: Vietnam; stateProvince: Nghe An; locality: Ky Son; verbatimElevation: 2,019 m; verbatimLatitude: 19°13.181’N; verbatimLongitude: 104°06.166’E

##### Conservation status

Least Concern

##### Distribution

Stripe-throated Yuhina has been recorded as a common resident in West Tonkin and the southern part of Central Annam (Robson 2008, Craik and Minh 2018, Hung et al. 2021b). This is the first confirmed record of the species for North Annam, Central Vietnam. Elsewhere, this species has been recorded as a resident in Bhutan, China, India, Laos, Myanmar and Nepal (IUCN 2024).

##### Diagnosis

**Figure 11.**
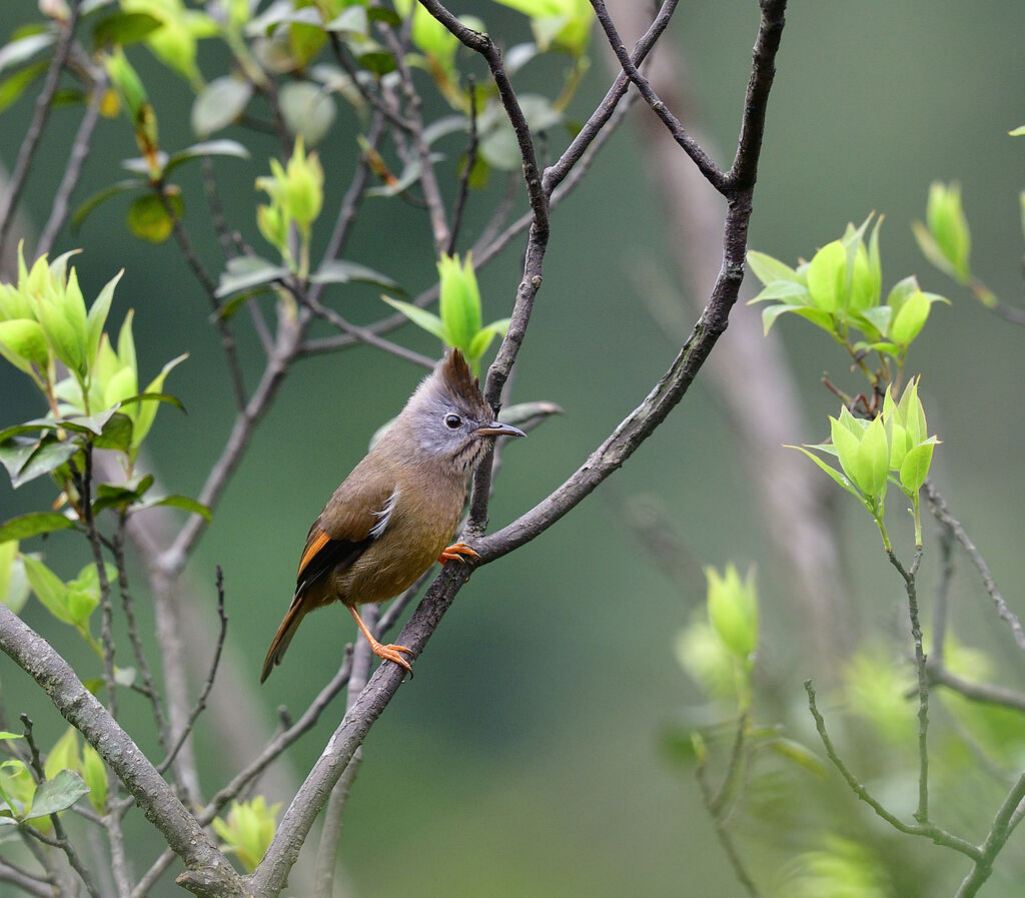
The adult *Yuhinagularis* (IEBR B.591) was photographed in the Ky Son area, Nghe An Province.

Size: 14-15.5 cm. **Adult**: Upperparts rather warm-tinged drab brown, greyish-pink breast, belly and vent deep ochre-buff with browner flanks and centres of undertail-coverts, primaries mostly blackish with some whitish fringing, tall forward-curved crest, pale pinkish to buffish throat (centre whiter) with blackish streaks, prominent pale orange-buff fringes to secondaries (Fig. 11). **Juvenile**: Slightly darker and warmer brown above, shorter crest.

#### Actinodura strigula

(Hodgson, 1837)

D47C43E4-B5AB-5507-9083-033E2CEC7795

https://www.gbif.org/species/8372464

- *Chrysominlastrigula*
- *Minlastrigula*

##### Materials

- **Type status:** Other material. **Occurrence:** occurrenceID: 6AD42126-DD30-5449-B5DD-EC72F7EED2AD; **Taxon:** kingdom: Animalia; phylum: Chordata; class: Aves; order: Passeriformes; family: Leiotrichidae; genus: Actinodura ; **Location:** country: Vietnam; stateProvince: Nghe An; locality: Ky Son; verbatimElevation: 2,005 m; verbatimLatitude: 19°13.238’N; verbatimLongitude: 104°06.192’E

##### Conservation status

Least Concern

##### Distribution

Bar-throated Minla has been recorded as a locally common resident in West Tonkin and the southern part of Central Annam (Robson 2008, Craik and Minh 2018, Hung et al. 2021b). This is the first confirmed record of the species for North Annam, Central Vietnam. Elsewhere, this species has been recorded as a resident in Bhutan, China, India, Laos, Malaysia, Myanmar, Nepal and Thailand (IUCN 2024).

##### Diagnosis

**Figure 12.**
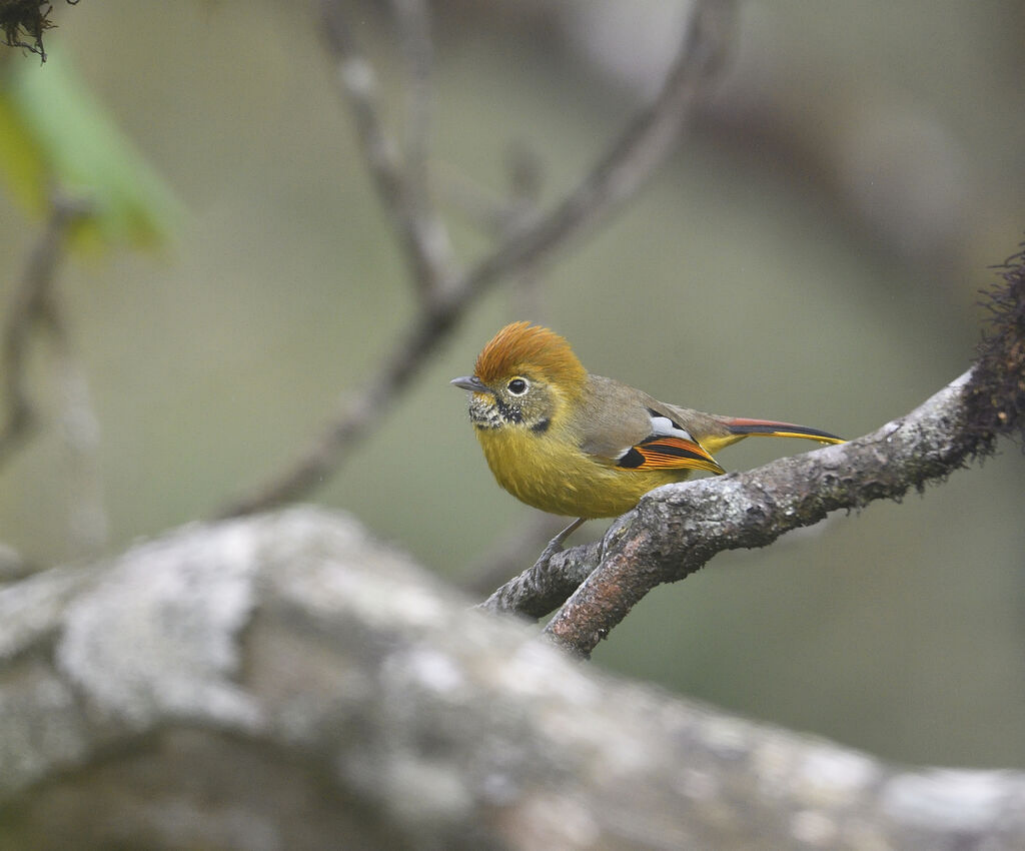
The adult *Actinodurastrigula* (IEBR B.598) was photographed in the Ky Son area, Nghe An Province.

Size: 16-18.5 cm. **Adult**: Golden-rufous crown, greyish head-sides, blackish eyebrow (to above eye), strong submoustachial streaks and mostly whitish throat with broad black bars/scales diagnostic. Olive-greyish upperparts, dull brownish-chestnut and black tail with whitish tip, yellowish underparts, black primary coverts, constrasting orange-yellow flight-feather fringes and broad white-bordered tertials (Fig. 12). **Juvenile**: Hind-crown, nape and upper parts greyer, paler head-sides, throat-bars narrower and more broken, underparts more washed out.

#### Plegadis falcinellus

Linnaeus, 1766

27BFB490-43B1-5056-A6FD-81A591BC9B2A

https://www.gbif.org/species/2480773

-*Tantalusfalcinellus*
-*Ibisfalcinellus*
-*Falcinellusfalcinellus*

##### Materials

- **Type status:** Other material. **Occurrence:** occurrenceID: 301C7A62-6B11-567C-9E0D-A99056E938CF; **Taxon:** kingdom: Animalia; phylum: Chordata; class: Aves; order: Pelecaniformes; family: Threskiornithidae; genus: Plegadis ; **Location:** country: Vietnam; stateProvince: Thua Thien Hue; locality: O Lau river; verbatimElevation: 1 m; verbatimLatitude: 16°40.109’N; verbatimLongitude: 107°24.361’E

##### Conservation status

Least Concern

##### Distribution

Glossy Ibis has been recorded as a vagrant in East Tonkin and a fairly common resident in Cochinchina (Robson 2008, Craik and Minh 2018, Hung et al. 2021b). This is the first confirmed record for the species in Central Annam. Globally, this species is widespread from Africa to southern Europe. In Southeast Asia, Glossy Ibis is recorded as a resident in Cambodia, Myanmar, Philippines and Thailand (IUCN 2024).

##### Diagnosis

**Figure 13.**
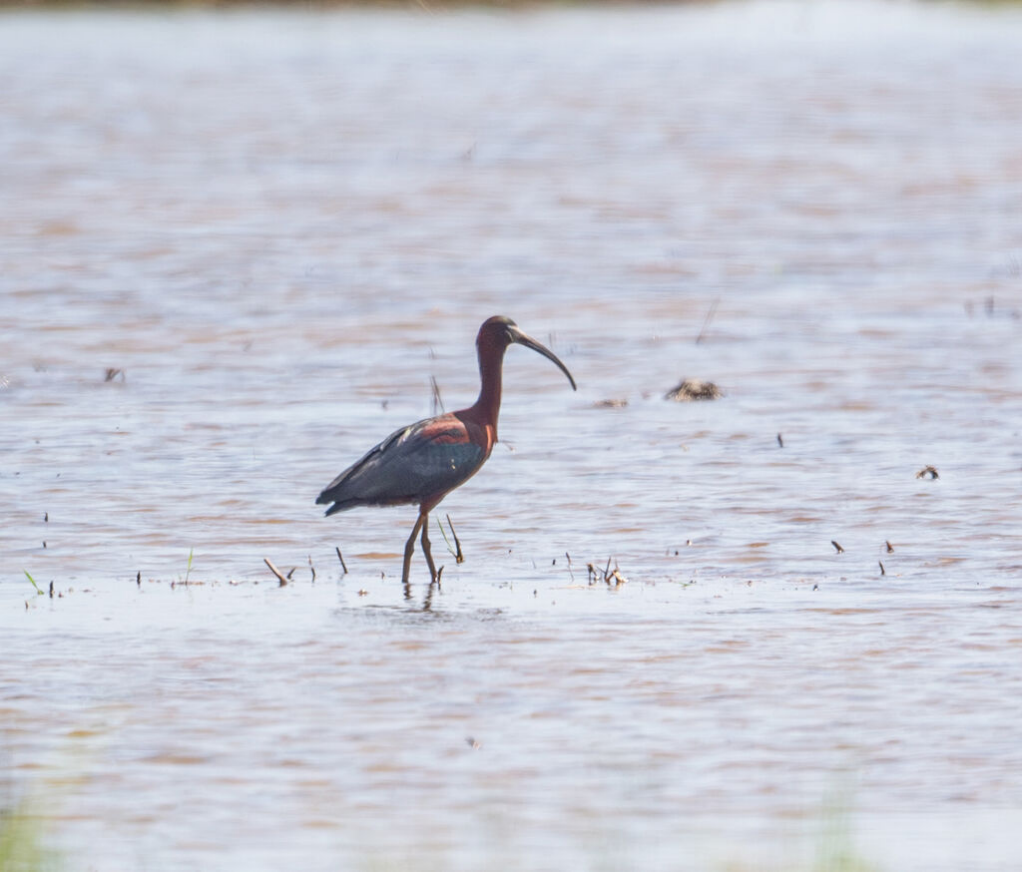
The adult *Plegadisfalcinellus* in breeding plumage (IEBR B.628) was photographed in O Lau rivermouth, Thua Thien Hue Province

Size: 55-65 cm. **Adult non-breeding**: Mostly uniform dark brownish with white streaks on head and neck, green gloss on scapulars and upperwing-coverts. Pale brownish bill, dark brownish legs and feet, dark facial skin bordered above and below by distinctive narrow white line. **Adult breeding**: Head, neck and body mostly deep chestnut, forecrown glossed green, much of plumage with purplish tinge, no streaks on head and neck, but more pronounced white border to lores, bill mostly flesh-coloured (Fig. 13).

#### Acrocephalus tangorum

La Touche, 1912

D0F80B9B-EB1E-5C2E-8331-D97C44D13BCA

https://www.gbif.org/species/5789158

Acrocephalus agricola tangorum

##### Materials

- **Type status:** Other material. **Occurrence:** occurrenceID: C34D6A85-B47C-5532-9546-963BBEC9CA83; **Taxon:** kingdom: Animalia; phylum: Chordata; class: Aves; order: Passeriformes; family: Acrocephalidae; genus: Acrocephalus ; **Location:** country: Vietnam; stateProvince: Khanh Hoa; locality: Thuy Trieu lake; verbatimElevation: 4 m; verbatimLatitude: 16°59.038’N; verbatimLongitude: 109°12.415’E

##### Conservation status

Vulnerable

##### Distribution

Manchurian Reed Warbler has been recorded as a scarce passage migrant in West Tonkin, East Tonkin and Cochinchina (Robson 2008, Craik and Minh 2018, Hung et al. 2021b). This is the first record of the species in South Annam. Manchurian Reed Warbler has recently been listed as Vulnerable by the IUCN Red List 2024. Elsewhere, this species has been recorded breeding in China, Myanmar and Russia and wintering in Cambodia, Laos, Malaysia, Peninsular and Thailand, with vagrant records in Korea (IUCN 2024).

##### Diagnosis

**Figure 14.**
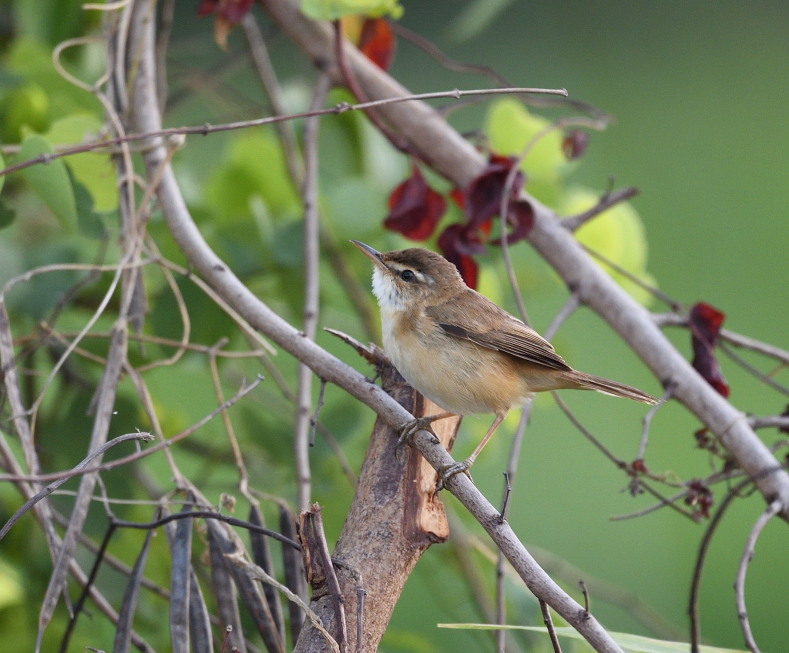
The adult *Acrocephalustangorum* (IEBR B.637) was photographed in Thuy Trieu Lake, Khanh Hoa Province.

Size: 13-14.5 cm. **Adult**: Crown and upperparts warm olive-brown with slighly more rufescent rump and uppertail-coverts, underparts whitish with warm buff breast-sides and flanks, whitish supercilium, narrow blackish line on crown-sides, long bill with pale lower mandible, white throat (Fig. 14).

#### Certhia manipurensis

Hume, 1881

B750B1FD-CDF3-5AB0-ABF2-3884C3C24AEA

https://www.gbif.org/species/5789209

Certhia manipurensis Hume, 1881

##### Materials

- **Type status:** Other material. **Occurrence:** occurrenceID: 78A647A5-3FE5-5347-97EE-AD06B3F11DE2; **Taxon:** kingdom: Animalia; phylum: Chordata; class: Aves; order: Passeriformes; family: Certhiidae; genus: Certhia ; **Location:** country: Vietnam; stateProvince: Nghe An; locality: Ky Son; verbatimElevation: 2,092 m; verbatimLatitude: 19°13.167’N; verbatimLongitude: 104°06.158’E

##### Conservation status

Least Concern

##### Distribution

Hume’s Treecreeper has been recorded as an uncommon to locally common resident in West Tonkin and South Annam (Robson 2008, Craik and Minh 2018, Hung et al. 2021b). This is the first confirmed record of the species for North Annam, Central Vietnam. Elsewhere, this species has been recorded as resident in India (Manipur), Laos, Myanmar and Thailand (IUCN 2024).

##### Diagnosis

**Figure 15.**
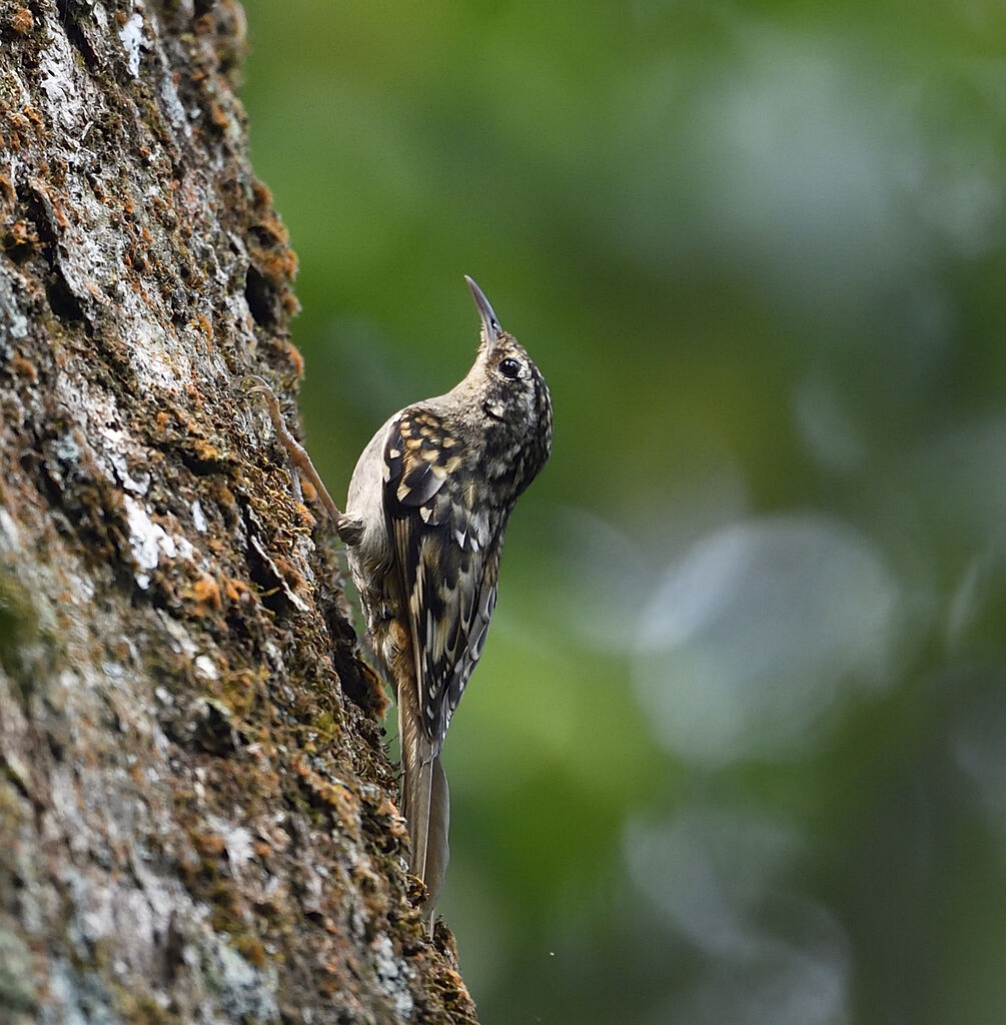
The adult of *Certhiamanipurensis* (IEBR B.675) was photographed in the Ky Son area, Nghe An Province.

Size: 15-16 cm. **Adult**: Brown upperparts with buff and white streaks, spots and mottling and an orange-brown rump, brown tail. Combination of drab greyish throat and underparts, buffy vent and indistinct supercilium diagnostic (Fig. 15). **Juvenile**: Shows faint darker scaling on throat and breast.

#### Sitta himalayensis

Jardine & Selby, 1835

47CFF200-F0BB-5282-8DF6-85EAA43B8438

https://www.gbif.org/species/2484888

Sitta himalayensis Jardine & Selby, 1835

##### Materials

- **Type status:** Other material. **Occurrence:** occurrenceID: 9CECB4A4-28E7-5279-9977-AE81F8E08075; **Taxon:** kingdom: Animalia; phylum: Chordata; class: Aves; order: Passeriformes; family: Sittidae; genus: Sitta ; **Location:** country: Vietnam; stateProvince: Nghe An; locality: Ky Son; verbatimElevation: 2,211 m; verbatimLatitude: 19°13.253’N; verbatimLongitude: 104°05.502’E

##### Conservation status

Least Concern

##### Distribution

White-tailed Nuthatch has been recorded as an uncommon resident in West Tonkin (Robson 2008, Craik and Minh 2018, Hung et al. 2021b). This is the first confirmed record of the species for North Annam, Central Vietnam. Elsewhere, this species has been recorded as a resident in Bhutan, China, India, Laos, Myanmar and Nepal (IUCN 2024).

##### Diagnosis

**Figure 16.**
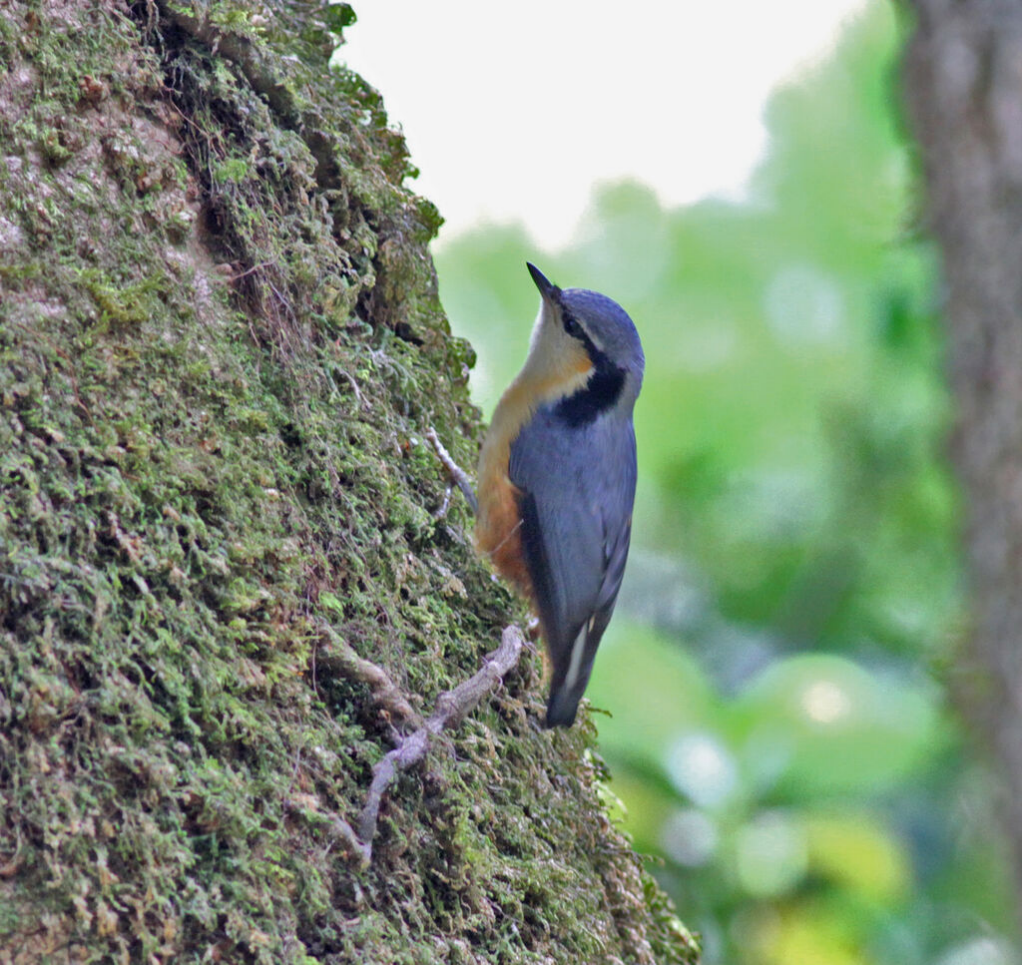
Male *Sittahimalayensis* (IEBR B.684) was photographed in the Ky Son area, Nghe An Province.

Size: 12 cm. **Male**: Grey upperparts with two black lines from eyes to lower nape, buff underside, white on the uppertail coverts, small bill and rufous-orange underparts with unmarked bright rufous undertail-coverts, whitish throat (Fig. 16). **Female**: Ear-coverts and underparts slightly paler and duller.

#### Sitta formosa

Blyth, 1843

093A7953-4E71-5023-A837-D343249A9408

https://www.gbif.org/species/2484903

Callisitta formosa

##### Materials

- **Type status:** Other material. **Occurrence:** occurrenceID: C822C806-BBA4-5A84-958B-F4BADA8FBCB8; **Taxon:** kingdom: Animalia; phylum: Chordata; class: Aves; order: Passeriformes; family: Sittidae; genus: Sitta ; **Location:** country: Vietnam; stateProvince: Nghe An; locality: Ky Son; verbatimElevation: 2,088 m; verbatimLatitude: 19°13.167’N; verbatimLongitude: 104°06.158’E

##### Conservation status

Vulnerable

##### Distribution

Beautiful Nuthatch has been recorded as a scarce to uncommon resident in West Tonkin and East Tonkin (Robson 2008, Craik and Minh 2018, Hung et al. 2021b). This is the first confirmed record of the species for North Annam, Central Vietnam. Beautiful Nuthatch was recently listed as Vulnerable in the IUCN Red List 2024. Elsewhere, this species has been recorded as a resident in Bhutan, China, India, Laos, Myanmar and Thailand (IUCN 2024).

##### Diagnosis

**Figure 17.**
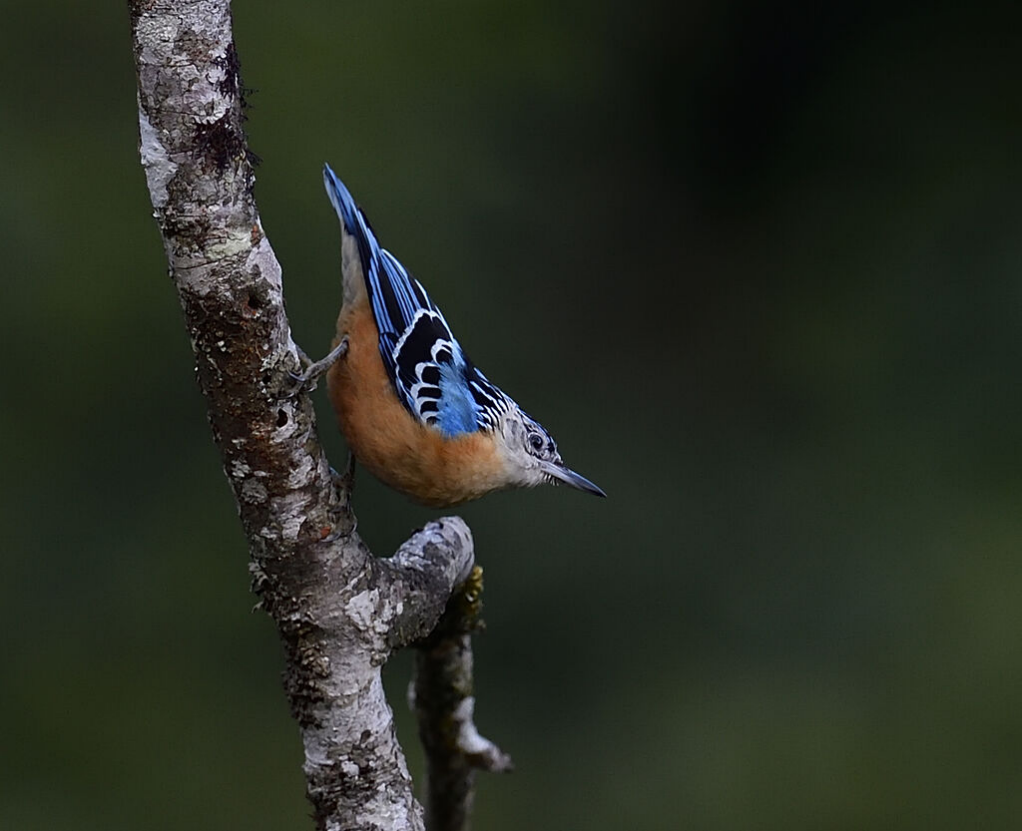
Adult *Sittaformosa* (IEBR B.691) was photographed in the Ky Son area, Nghe An Province.

Size: 16.5 cm. **Adult**: Large, upperparts black, streaked brilliant blue to white on crown, nape and mantle, broad blue band along scapulars to back and rump, black wings with two narrow white wing-bars, dull rufous-buff underparts with paler throat and head-sides (Fig. 17). **Juvenile**: Very similar to adult, but white streaks on upperparts may be bluer, whiter and paler underparts, particularly on breast.

#### Cutia nipalensis

Hodgson, 1837

AF02B26E-794B-5DA2-8F87-7C4E62663E2A

https://www.gbif.org/species/5231396

Cutia nipalensis nipalensis

##### Materials

- **Type status:** Other material. **Occurrence:** occurrenceID: 782DEADB-6B0A-53D4-AA87-19DCC6D2D223; **Taxon:** kingdom: Animalia; phylum: Chordata; class: Aves; order: Passeriformes; family: Leiotrichidae; genus: Cutia; **Location:** country: Vietnam; stateProvince: Nghe An; locality: Ky Son; verbatimElevation: 2,225 m; verbatimLatitude: 19°14.043’N; verbatimLongitude: 104°05.126’E

##### Conservation status

Least Concern

##### Distribution

Himalayan Cutia has been recorded as a scarce to fairly common resident in West Tonkin (Robson 2008, Craik and Minh 2018, Hung et al. 2021b). This is the first confirmed record of the species for North Annam, Central Vietnam. Elsewhere, this species has been recorded as a resident in Bhutan, China, India, Malaysia, Myanmar, Nepal and Thailand (IUCN 2024).

##### Diagnosis

**Figure 18.**
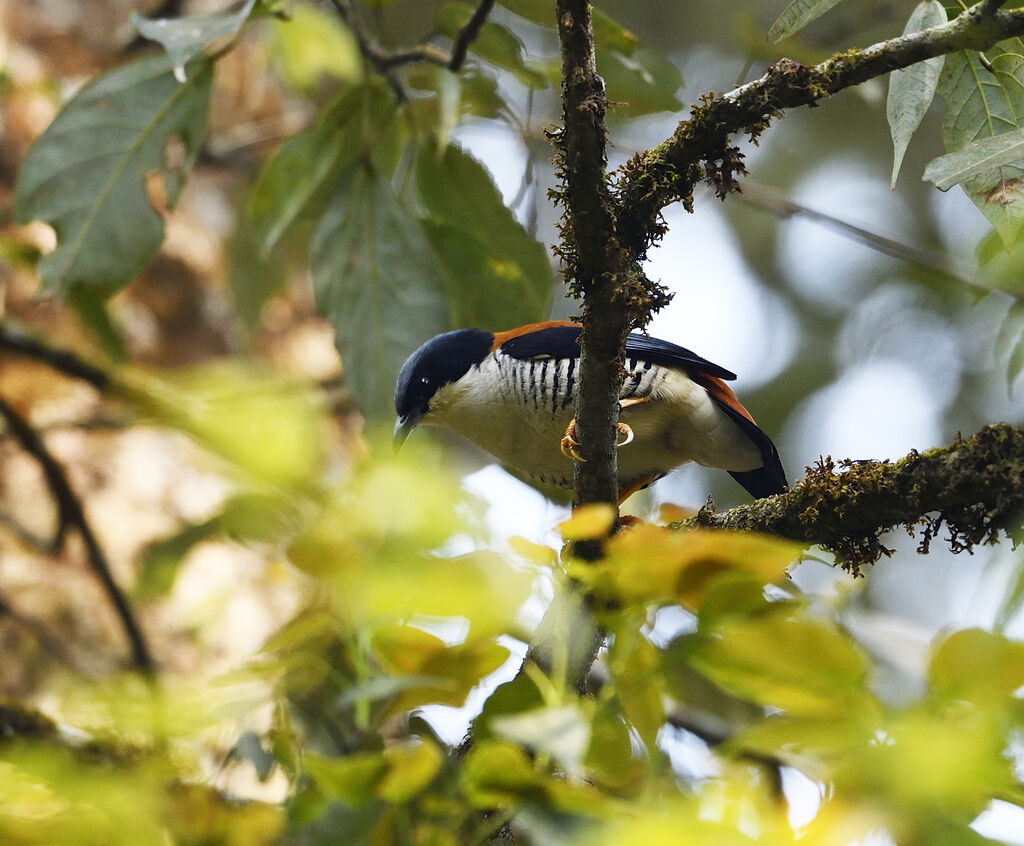
Male *Cutianipalensis* (IEBR B.698) was photographed in the Ky Son area, Nghe An Province.

Size: 17-19.5 cm. **Male**: Bluish-slate crown, black head-sides, rufous-chestnut rest of upperparts, whitish underparts with bold black bars on breast-sides and flanks, wings black with tertials and flight-feathers fringed bluish-grey, flanks and vent washed buff (Fig. 18). **Female**: Like male, but mantle, back and scapulars more olive-brown with broad blackish streaks, dark brown head-sides. **Juvenile**: Duller than respective adults with browner crown and fainter dark bars on underparts.

#### Emberiza lathami

Gray, JE, 1831

6299D19D-4874-5970-9D6B-DF5380D2E119

https://www.gbif.org/species/6100799

Melophus lathami

##### Materials

- **Type status:** Other material. **Occurrence:** occurrenceID: AEFA0C7D-36C7-5ACE-8640-23536E631445; **Taxon:** kingdom: Animalia; phylum: Chordata; class: Aves; order: Passeriformes; family: Emberizidae; genus: Emberiza ; **Location:** country: Vietnam; stateProvince: Nghe An; locality: Ky Son; verbatimElevation: 532 m; verbatimLatitude: 19°17.024’N; verbatimLongitude: 104°10.492’E

##### Conservation status

Least Concern

##### Distribution

Crested Bunting has been recorded as an uncommon to locally common resident in West Tonkin and East Tonkin (Robson 2008, Craik and Minh 2018, Hung et al. 2021b). This is the first confirmed record of the species for North Annam, Central Vietnam. Elsewhere, this species has been recorded breeding in Bhutan, Bangladesh, China, India, Laos, Myanmar, Nepal, Pakistan and Thailand (IUCN 2024).

##### Diagnosis

**Figure 19.**
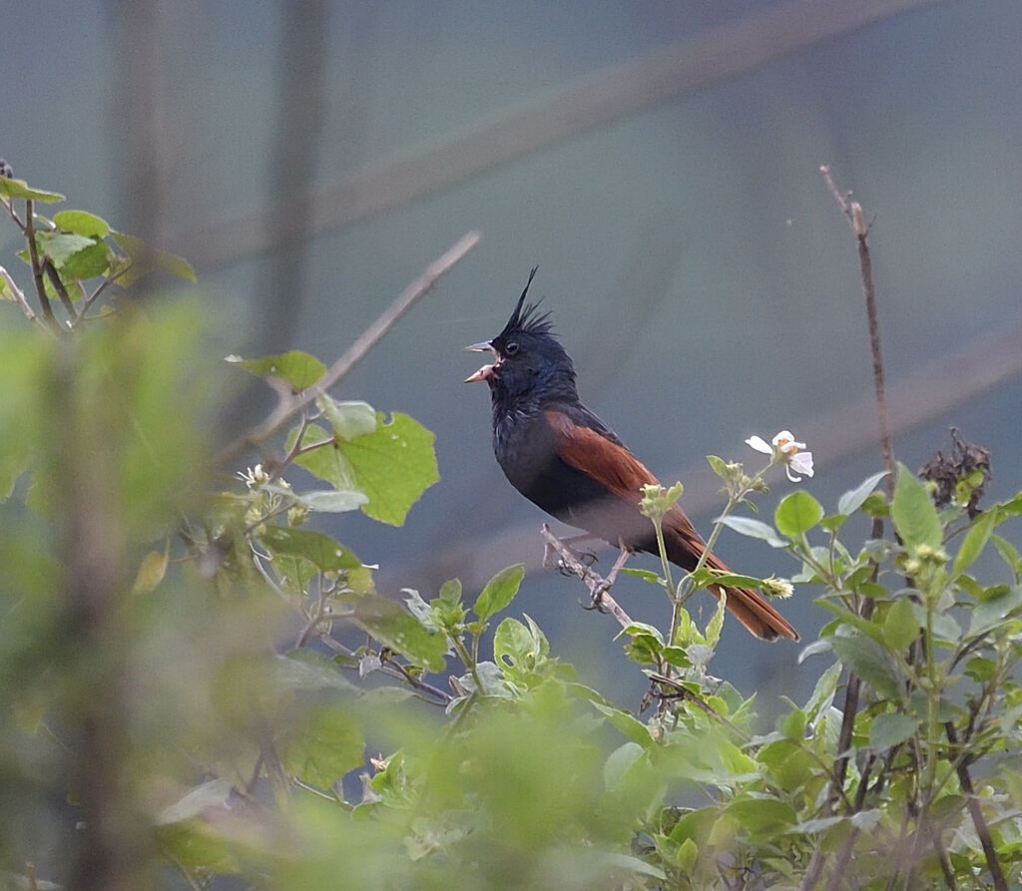
Male breeding *Emberizalathami* (IEBR B.708) was photographed in the Ky Son area, Nghe An Province.

Size: 16.5-17 cm. **Male breeding** : Blackish head and body, chestnut wings and tail, long erect crest (Fig. 19). **Male non-breeding**: Blackish body feathers edged buffish-grey. **Female breeding**: Much paler and browner with less chestnut wings and tail, shorter crest. Upperpart olive-brown, streaked darker, underparts paler with faint dark breast streaking. Largely chestnut wings and tail; short crest and lack of white on tail rules out other buntings. **Female non-breeding**: A little darker than breeding, mantle sandy-brown and more diffusely streaked. **Juvenile**: Like female, but slightly darker with shorter crest, underparts buffier with more extensive, but fainter breast streaking. Male develops black blotching on body plumage.

## Discussion

The documentation of new distribution data for 15 bird species in Central Vietnam has shown that the region still holds a high potential to discover additional species diversity.

The central coastal region of Vietnam is located along the East Asia – Australia Flyway. Collecting information related to migratory and wintering bird species is necessary and urgent to provide comprehensive information on the status of migratory species as well as migratory routes, which would help decision-makers with wildlife conservation planning for a better conservation of existing sensitive habitats (mudflats, marshes, reedbeds).

Many of the bird species recorded in the Ky Son area of Nghe An Province have the centre of their distribution in the Himalayan range (Robson 2008, Craik and Minh 2018, Hung et al. 2021b). In Vietnam, the easternmost extensions of the Himalayan range end at the Hoang Lien Mountain of Lao Cai Province (about 500 km away from the Ky Son area). This shows that the area forms an important transition between the Himalayan and Annam mountain ranges.

Our surveys also brought to light many impacts on bird species including habitat destruction and development projects in a number of wetland areas (Suppl. material 1), particularly at the Tra O and Thuy Trieu Lakes (Binh Dinh and Khanh Hoa Provinces). Hunting, trapping and bird trade still occur regularly (Suppl. material 2), especially impacting waterfowl during migration seasons and targeting songbirds for the pet trade. This raises an alarming situation and requires immediate treatment and management measures to preserve wild birds and their habitats in Vietnam.

## Supplementary Material

XML Treatment for Fregata ariel

XML Treatment for Emberiza pusilla

XML Treatment for Pycnonotus xanthorrhous

XML Treatment for Alcurus striatus

XML Treatment for Spizixos canifrons

XML Treatment for Saxicola jerdoni

XML Treatment for Yuhina gularis

XML Treatment for Actinodura strigula

XML Treatment for Plegadis falcinellus

XML Treatment for Acrocephalus tangorum

XML Treatment for Certhia manipurensis

XML Treatment for Sitta himalayensis

XML Treatment for Sitta formosa

XML Treatment for Cutia nipalensis

XML Treatment for Emberiza lathami

3286788B-E108-5DF7-B9A1-C0BFBBE92D79 Supplementary material 1 Conversion of coastal wetlands to build the new road and bridges in Central Vietnam Data type Images File: oo_1112149.jpg https://binary.pensoft.net/file/1112149 L M Hung

77078ED9-F10A-5D4E-BCC1-37AA4A64E18C 10.3897/BDJ.12.e133721.suppl2 Supplementary material 2 Trading of migratory birds at the Central Vietnam Data type Images File: oo_1112153.jpg https://binary.pensoft.net/file/1112153 L M Hung
