# Supplementary figures and images for: New records of birds from Central Vietnam
Source: Biodivers Data J. 2024 Aug 20;12:e133721. doi: 10.3897/BDJ.12.e133721 (PMC11350280; doi:10.3897/BDJ.12.e133721)

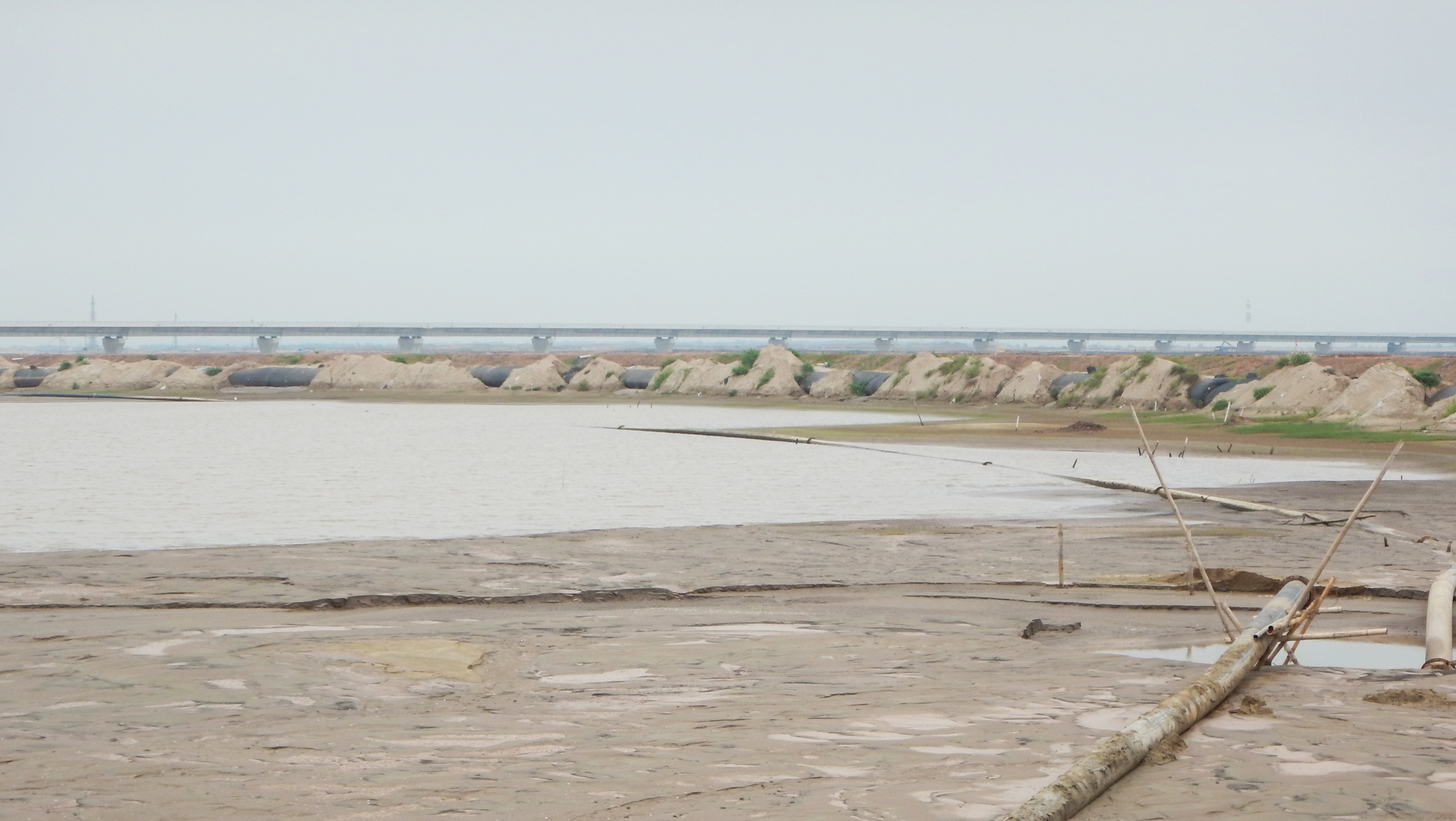

Supplement: Supplementary material 1 — Conversion of coastal wetlands to build the new road and bridges in Central Vietnam [file bdj-12-e133721-s001.jpg]

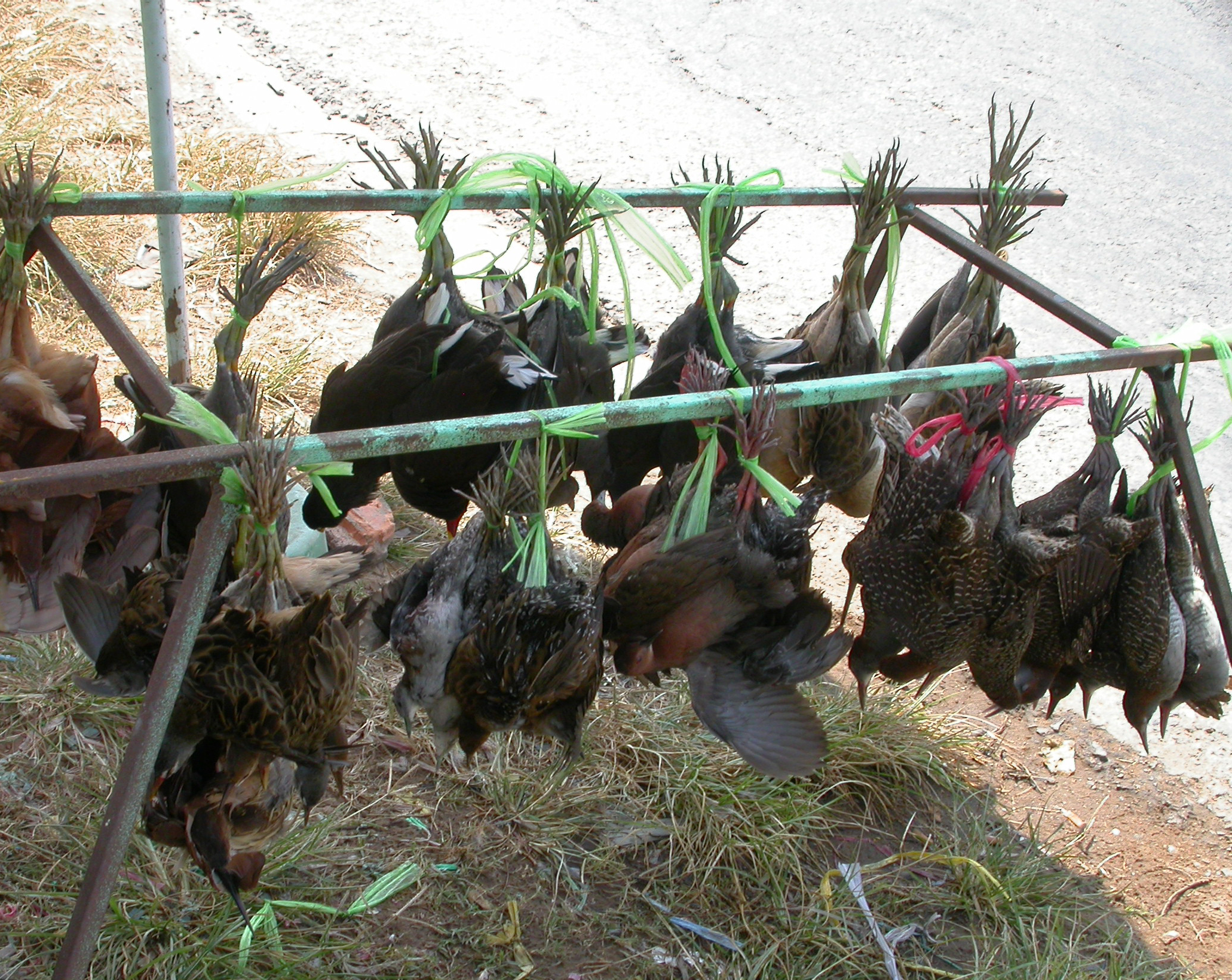

Supplement: Supplementary material 2 — Trading of migratory birds at the Central Vietnam [file bdj-12-e133721-s002.jpg]
